# Decrease of resistance to air flow with nasal strips as measured with the airflow perturbation device

**DOI:** 10.1186/1475-925X-3-38

**Published:** 2004-10-22

**Authors:** Lily S Wong, Arthur T Johnson

**Affiliations:** 1 Biological Resources Engineering, University of Maryland, College Park, MD, 20742, USA; 2 Biological Resources Engineering, University of Maryland, College Park, MD, 20742, USA

## Abstract

**Background:**

Nasal strips are used by athletes, people who snore, and asthmatics to ease the burden of breathing. Although there are some published studies that demonstrate higher flow with nasal strips, none had directly measured the effect of the strips on nasal resistance using the airflow perturbation device (APD). The APD is an inexpensive instrument that can measure respiratory resistance based on changes in mouth pressure and rate of airflow.

**Method:**

This study tested forty-seven volunteers (14 men and 33 women), ranging in age from 17 to 51. Each volunteer was instructed to breathe normally into the APD using an oronasal mask with and without nasal strips. The APD measured respiratory resistance during inhalation, exhalation, and an average of the two.

**Results:**

Results of a paired mean t-test comparing nasal strip against no nasal strip were statistically significant at the p = 0.05 level. The Breathe Right™ nasal dilator strips lowered nasal resistance by an average of 0.5 cm H_2_0/Lps from an average nasal resistance of 5.5 cm H_2_0/Lps.

**Conclusions:**

Nasal strips reduce nasal resistance when measured with the APD. The effect is equal during exhalation and during inhalation.

## Background

Nasal dilator strips (NDS) are used by athletes, people who snore, and asthmatics to ease the burden of breathing. The nasal strips are used as a mechanical means of reducing nasal airflow resistance [1]. By lowering nasal resistance, they reduce the work of breathing and the supply of oxygen into the body could increase [2,3].

The size of the nostril limits the amount of air entering into the body. The NDS is placed along the nasal valve of the nose. The adhesiveness of the strip binds to the creases of the nasal valve to prevent the outer wall tissue of the nose from collapsing inward during nasal breathing. This mechanism thus dilates the nose and allows more air to flow into the nose [3].

The primary effect of the NDS could be either to dilate the air passage of the nose or to stiffen the nasal wall. Either mechanism would reduce nasal resistance and allow higher flow of air, but they can be distinguished over a range of air flows. Stiffening the nasal wall would have its most profound effect at higher flows where the Bernoulli effect would decrease internal nasal pressures and tend to constrict nasal passage diameter. Air passage dilation would tend to decrease nasal resistance more uniformly over a range of air flows.

Recent studies on the effectiveness of Breathe Right™ nasal strips tested participants under various rest and exercise conditions. Some found that the strips neither improve or diminish airflow [2,4-9], which contradict results found by others [1,3,10-14]. Various techniques were used to assess NDS effectiveness. Some measured the amount of airflow, others the area of the nostrils, and still others the nasal airflow resistance.

**Figure 1:**
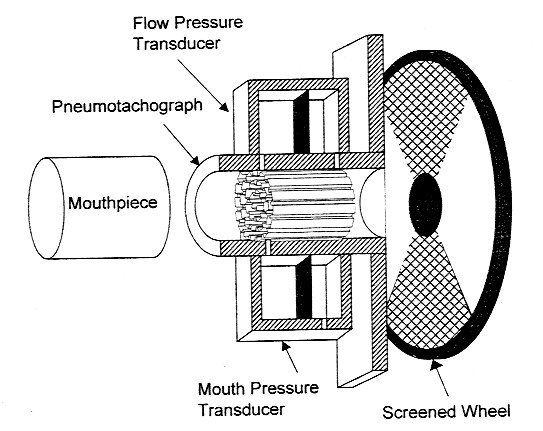
The APD Sensor consists of a rotating wheel in the air path, a pneumotach, and pressure transducers [15]

**Figure 2:**
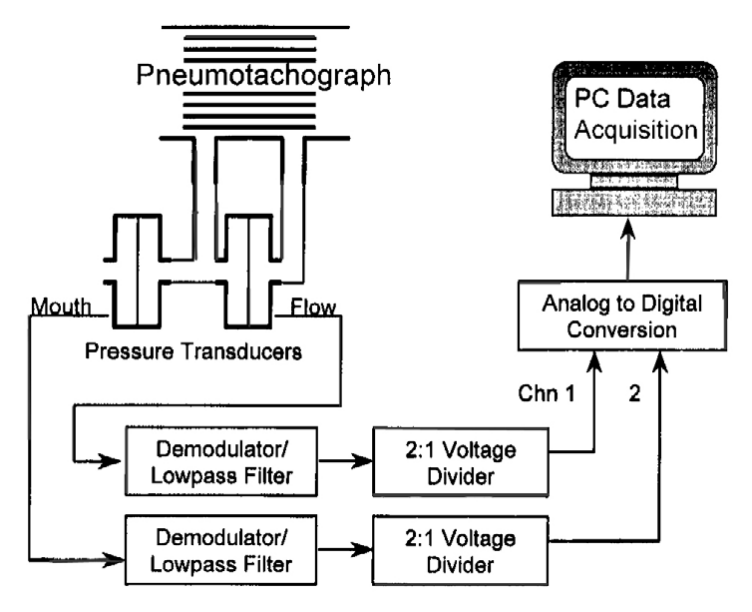
The APD system consists of pressure and flow transducers, analog-to-digital conversion, and a computer display of results [1].

The Airflow Perturbation Device (APD) is a small, light weight, and easy to use instrument that measures respiratory resistance [15]. A segmented rotating wheel in the air flow path changes air flow and mouth pressure as the wheel momentarily partially obstructs the flow passage (Figure 1). The magnitude of these perturbations depends on the resistance of the wheel and respiratory resistance. Measurement of wheel resistance allows respiratory resistance to be calculated directly (Figure 2). Resistance values appear on a computer screen within a minute from starting the measurement. Thereafter they are updated as they occur.

People breathe normally through the APD. No special breathing maneuvers are required. For this reason, the APD can be used with young children, older adults, unconscious patients, and animals. Respiratory resistance can be separated into inhalation and exhalation components, and resistance can be displayed against lung volume and air flow rate.

Respiratory resistance is normally measured through the mouth, with a nose clip and hands pressed against the cheeks. An oronasal mask may be used to obtain combined mouth and nose resistance, or nose resistance by itself if the mouth is closed. The APD used with an oronasal mask should be an ideal instrument to assess the effect of NDS.

This study is as much a test of the capabilities of the APD as it is a study of NDS. Objectives of this study were to: 1) determine if APD measurements of respiratory resistance measured with an oronasal mask matched those with breathing through a mouthpiece, and 2) measure the effects of NDS on respiratory resistance made with the APD. This is not a clinical study.

## Methods

This study tested forty-seven volunteers (14 men, 33 women; age 17–51 yr; height 147–188 cm; weight 38–105 kg). Some had symptoms of nasal congestion such as asthma, allergies, and snoring. A written informed consent was obtained from each subject and the protocol was approved by the University of Maryland Institutional Review Board (IRB).

The nasal strips used in this study were a commercial product called "Breathe Right" (CNS, Inc., Minneapolis, MN, clear, medium/large nasal strips). According to the manufacturer's instructions, the nasal strips should be placed halfway down the nose along the nasal valve. The two end regions of the nasal strips should cover the left and right nasal creases.

This study contained three phases: 1) to determine the ability of the APD to measure oral resistance using either a mouthpiece or an oronasal mask, 2) to determine the ability of the APD to measure nasal resistance, and 3) to determine the effect of nasal strips on nasal resistances. Phase I consisted of two tests that measured oral breathing resistance. In the first test, the subject's nose was occluded with two layers of Durapore surgical tape (3 M, St. Paul, MN) while the subjects were sitting in an upright position. The subject was instructed to breathe normally into a cardboard mouthpiece. The second test repeated the same procedure as the first test, except the subject was breathing into an oronasal mask (Adult Mask 4–5^+^, Laerdal Medical, Wappingers Falls, NY). The subject was instructed to press their face against the mask while he/she breathed normally. The size of the mask was large enough that it contacted only the hard tissue on the bridge of the nose and did not compress the soft nasal septum.

The second phase of this study consisted of a test to measure nasal breathing resistance. The subject was instructed to breathe normally through the nose with the mouth closed and with no NDS. For the third phase, the subject placed a NDS across the nasal valve on his/her nose as shown on the instructions provided by the manufacturer. In both of these tests, the oronasal mask was used.

Air flow perturbations with the APD occur at a rate of about 10 per second [15]. Measurements were obtained in these experiments over approximately 100 perturbations. It has been previously found that measurements made over that time are relatively stable and reproducible [15]. Several time-averaged resistance values are displayed: resistance during inhalation, 2) resistance during exhalation, and 3) the average of inhalation and exhalation resistances. All three of these have been found to be useful.

Primary comparisons for this study were made using the average respiratory resistance. Secondary comparisons in the second phase of this study investigated the effects of NDS on inhalation and exhalation respiratory resistances. Statistical comparisons were made using a paired mean t-test with significance at the p = 0.05 level.

## Results

Subject data appear in Table 1. Average resistance measured during mouth breathing with mouthpiece ranges from 1.90 to 5.03 cm H_2_O/Lps. In the past, average respiratory resistances for healthy adults have generally fallen in the range of 2.5 - 3.5 cm H_2_O/Lps, and such is the case here. Also, as expected, most respiratory resistance values during exhalation exceed those measured during inhalation.

**Table 1:** Subject data for APD Measurements of Respiratory Resistance when Measured Through the Mouth and Nose. Resistances are given in cm H_2_O/Lps.

**Subject No.**	**Sex**	**Mouth Piece**			**Mask Mouth**			**Mask Nose**			**Mask NDS**		
	
		Inh	Avg	Exh	Inh	Avg	Exh	Inh	Avg	Exh	Inh	Avg	Exh
1	F	3.15	3.06	2.97	2.86	3.08	3.30	4.65	5.13	5.61	4.08	4.13	4.18
2	F	3.00	3.26	3.52	2.98	3.29	3.59	5.84	5.72	5.59	3.64	3.47	3.30
3	F	2.69	3.19	3.68	2.84	3.38	3.92	4.94	5.73	6.52	4.55	5.26	5.97
4	F	4.00	4.62	5.25	3.97	4.46	4.95	5.40	5.87	6.31	5.11	5.45	5.79
5	F	3.13	4.04	4.95	3.58	4.11	4.63	5.33	5.59	5.85	4.62	5.30	5.98
6	M	2.14	2.41	2.69	2.10	2.34	2.58	3.76	4.15	4.53	3.71	4.16	4.61
7	F	2.73	3.15	3.85	2.43	3.11	3.79	3.92	4.16	4.39	3.54	3.62	3.69
8	M	2.03	2.33	2.64	2.12	2.24	2.36	4.32	4.50	4.69	3.65	3.85	4.06
9	M	1.87	1.96	2.06	1.83	1.82	1.80	3.16	3.53	3.91	3.26	3.47	3.68
10	M	3.49	3.95	4.41	3.27	3.90	4.52	5.96	6.27	6.57	6.13	6.20	6.28
11	F	2.89	2.97	3.04	2.76	2.91	3.07	7.59	7.33	7.07	7.48	7.05	6.61
12	F	3.67	4.24	4.82	4.18	4.38	4.58	6.36	7.14	7.92	6.75	6.82	6.90
13	M	2.20	2.54	2.89	2.13	2.49	2.84	5.27	5.50	5.73	4.80	5.04	5.27
14	F	3.08	2.98	2.88	2.98	3.07	3.17	5.12	5.25	5.38	5.34	5.36	5.38
15	M	2.37	2.45	2.53	2.64	2.77	2.90	5.44	5.63	5.82	5.33	5.57	5.81
16	M	3.48	3.58	3.68	3.16	3.47	3.77	5.71	5.30	4.90	4.71	4.88	5.04
17	M	2.40	2.85	3.30	2.22	2.74	3.26	4.54	4.90	5.25	4.38	4.70	5.07
18	F	2.96	2.96	2.95	2.95	3.04	3.12	6.84	7.19	7.54	6.71	6.89	7.07
19	M	2.00	2.30	2.59	2.54	2.10	1.67	3.82	4.25	4.68	3.20	3.39	3.57
20	F	2.40	2.85	3.30	2.81	2.77	2.73	4.12	4.16	4.20	3.61	3.73	3.85
21	F	4.22	4.20	4.17	4.34	4.42	4.49	6.76	6.97	7.10	6.36	6.75	7.14
22	F	2.73	3.12	3.50	3.02	3.12	3.22	6.60	6.74	6.89	5.90	6.08	6.26
23	F	4.87	5.03	5.19	4.49	4.96	5.42	5.96	6.23	6.70	5.06	5.30	5.54
24	F	4.38	4.79	5.20	4.41	4.64	4.87	6.35	6.68	7.01	5.70	6.05	6.39
25	F	2.61	3.02	3.44	2.51	2.94	3.36	3.98	4.03	4.08	3.56	3.42	3.28
26	M	2.12	2.42	2.71	1.80	2.26	2.71	5.87	5.84	5.81	5.31	4.38	5.44
27	F	3.22	3.77	4.31	3.48	3.58	3.68	4.51	4.76	5.01	4.24	4.58	4.93
28	F	2.85	3.01	3.17	2.74	2.94	3.14	4.71	4.64	4.56	3.57	4.17	4.76
29	F	2.84	3.26	3.67	3.04	3.39	3.75	4.94	5.56	6.18	4.40	5.04	5.68
30	F	2.76	3.08	3.40	2.81	3.05	3.29	6.24	6.40	6.56	5.57	5.76	5.94
31	F	2.90	3.21	3.51	2.68	2.88	3.09	4.71	4.88	5.04	5.03	5.16	5.29
32	M	1.76	1.90	2.03	1.54	1.87	2.20	4.02	4.49	4.97	4.00	4.18	4.35
33	F	3.65	3.94	4.23	3.76	3.92	4.09	4.90	5.30	5.71	4.90	5.26	5.61
34	M	1.94	2.15	2.36	1.97	2.17	2.36	5.19	4.90	4.61	4.37	4.36	4.36
35	F	2.78	2.93	3.09	2.81	3.05	3.29	3.81	4.45	5.10	3.75	4.20	4.65
36	F	5.21	5.09	4.97	4.46	4.81	5.71	4.81	5.19	5.56	4.00	4.04	4.08
37	F	3.18	3.44	3.69	3.02	3.34	3.66	5.49	5.70	5.91	5.50	5.58	5.65
38	F	2.77	3.48	4.20	2.62	3.31	4.00	6.70	6.60	6.51	5.97	6.12	6.26
39	F	3.66	3.57	3.48	3.53	3.54	3.55	7.62	7.73	7.83	6.94	7.33	7.72
40	F	2.57	3.05	3.52	2.58	2.97	3.36	3.64	4.13	4.63	3.58	3.78	3.98
41	F	3.76	3.74	3.71	3.17	3.68	4.18	4.70	5.11	5.51	4.13	4.54	4.95
42	M	3.27	3.39	3.58	3.43	3.61	3.80	6.08	6.44	6.79	5.90	6.25	6.60
43	F	3.27	3.65	4.03	3.89	3.68	4.47	4.47	4.78	5.09	3.69	3.98	4.27
44	M	2.23	2.51	2.79	2.17	2.34	2.51	6.48	6.42	6.35	5.87	6.01	6.15
45	F	2.09	2.95	3.02	2.98	3.07	3.16	6.10	6.65	7.20	5.86	6.20	6.55
46	F	2.98	3.12	3.26	3.15	3.23	3.30	5.70	5.65	5.60	4.57	4.68	4.78
47	F	2.67	2.64	2.61	2.56	2.56	2.57	4.35	4.74	5.12	3.29	3.64	3.99
Average		2.96	3.24	3.51	2.96	3.21	3.48	5.24	5.50	5.74	4.80	5.00	5.25
Std dev		0.76	0.75	0.82	0.73	0.76	0.88	1.09	1.02	1.03	1.11	1.10	1.12

Breathing through the mouth into the oronasal mask yielded almost the same values. Means of values with the mouthpieces and oronasal mask are 3.24 and 3.21, respectively. The difference was not statistically significant using a paired-t test at p = 0.05.

**Figure 3:**
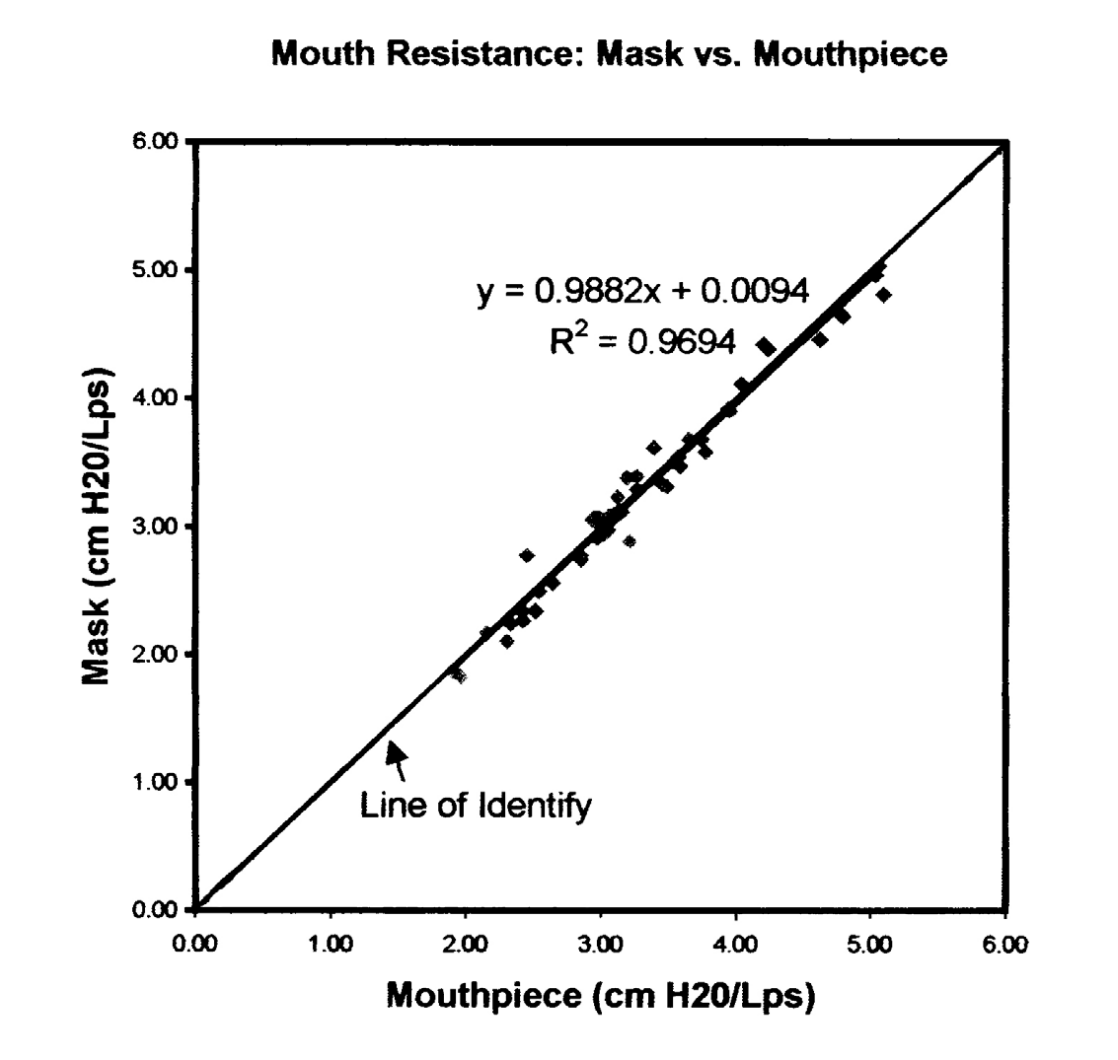
Average oral respiratory resistance measured with a mouthpiece and an oronasal mask. There is almost perfect agreement between the two methods.

Figure 3 shows the graph of average respiratory resistance of mask vs. mouthpiece while breathing through the mouth. The graph has a slope of nearly 1.0 and an intercept of nearly 0.0, indicating a nearly perfect correspondence between the two methods of measurement. Both slope and intercept were tested statistically and the line was found to be identical to y = x at the p = 0.05 level. Comparison of inhalation resistance between mouthpiece and oronasal mask yielded the following equation:

y = 0.9624 + 0.1041 R^2 ^= 0.8435    (1)

where y = mask value of resistance and x = mouthpiece value of resistance

This equation was tested to be statistically equivalent to y = x. This indicates that the oronasal mask had no effect on the inhalation values.

A similar comparison of exhalation resistances gave the following:

y = 0.874bx + 0.4594 R^2 ^= 0.8828    (2)

This equation did not pass the statistical test for equivalence y = x. The oronasal mask may have affected the measurement of respiratory resistance in the exhalation direction.

**Figure 4:**
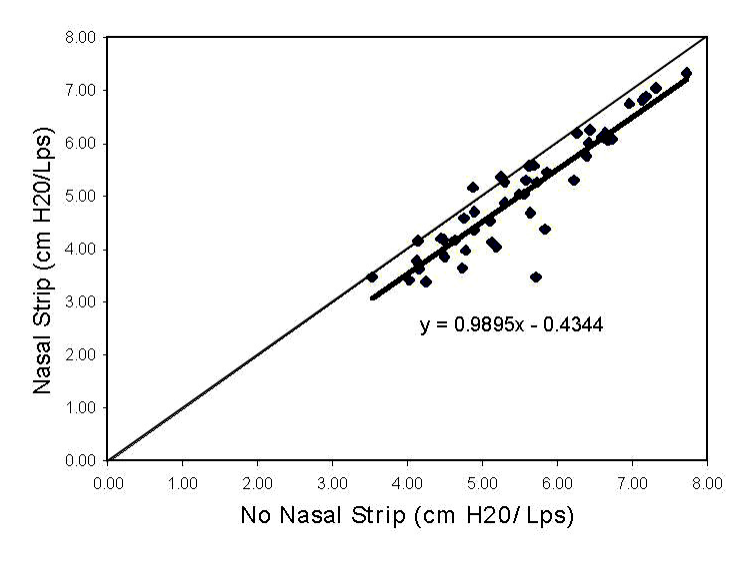
Average respiratory resistance while breathing through the nose in 47 subjects. Nasal strips showed a decrease of nasal resistance of 0.43 cm H_2_O/Lps.

Figure 4 shows the relationship of average respiratory resistance when breathing through the nose measured with and without the nasal strips. The NDS data have a slope of nearly 1.0 and a y-intercept is approximately -0.4. This signifies a reduction of nasal breathing resistance using the nasal strip. Nasal resistances with no nasal strip range from 3.53 to 7.73 cm H_2_O/Lps, while nasal resistance with the NDS ranges from 3.39 to 7.33 cm H_2_O/Lps. This demonstrates the expected resistance reduction with NDS.

All subjects except three showed a decrease in nasal resistance when breathing with the NDS. Average value of nose breathing without NDS was 5.50; with NDS it was 5.00. These means were highly statistically significantly different.

The effect of NDS on resistance during exhalation was also statistically highly significant. There was an average reduction of 0.45 cm H_2_O/Lps in resistance, and only six out of 47 subjects failed to demonstrate a decrease in resistance with NDS.

The effect of NDS on resistance during inhalation tested to be statistically highly significant, as well. The average resistance reduction was 0.49 cm H_2_0/dps. Again, six subjects failed to demonstrate a decrease in resistance with NDS. These were not the same subjects that increased resistance in the exhalation direction.

Resistance differences with and without NDS in the inhalation and exhalation directions were tested to determine if NDS had a larger effect while breathing in one direction or the other. Means of the differences for inhalation and exhalation directions were tested with a paired t-test, and found to be statistically nonsignificant. It appears, therefore, that NDS affect nasal resistances equally during inhalation and exhalation.

## Discussion

This study confirmed the results of other studies that showed a reduction of about 10% in nasal breathing resistance, as well as supported the claim of the manufacturer that the nasal strips provide nasal relief. Several subjects who had nasal congestion reported some relief in nasal breathing when using the nasal strip. Exactly which subjects these were was not recorded.

There was one surprise, though, in the results. The reduction in respiratory resistance due to NDS was a constant amount and not proportional to the resistance level present without NDS. This result was not expected, and no adequate explanation for it can be given at this time. It is not clear why this should be so, but we do not doubt that measurements made with the APD are correct, based on previous studies [15,16].

We cannot comment on the clinical significance of the resistance reduction with NDS use. It seems likely that some benefit could be obtained from such a resistance change, but whether it is actually detectable is not clear. Other reports in the literature [17,18] have concluded that the minimum detectable external resistance is about a constant 25–30% proportion of the resistance already present. The resistance change measured in this study is about 10% of the baseline resistance. If the use of NDS does result in a detectable change, then it may be that a different detection mechanism is operating. It is possible that the subject could detect nasal resistance only, rather than total respiratory resistance. Based on that supposition, NDS reduce nasal resistance by about 17%.

The APD has been shown to be able to measure respiratory resistance with either a mouthpiece or an oronasal mask. This may be a significant advantage of the instrument, especially because respiratory resistance measurement on unconscious or uncooperative patients would be much more easily made with a mask than with a mouthpiece. Equations (1) and (2) show the close correspondence between measurements made with both techniques, although the presence of an intercept and a slope different from unity indicate that the correspondence between mask and mouthpiece is not perfect. Resistances with a mask are both higher than resistances with a mouthpiece. The reason for this seems to be different mouth positions in both cases. We have laboratory experiences (not published) that demonstrate that tongue position can influence measured resistance. Breathing through the mask is probably done with the mouth closed more than when breathing through the mouthpiece. The measured difference between inhalation and exhalation resistances could reflect the effect of a pressure difference across the distensible smaller airways, which is greater inside than outside during inhalation, but smaller inside than outside during exhalation. This would lead to a dynamic compression of the small intrathoracic airways during exhalation. Another possible explanation is natural movement of the vocal chords such that they are closer during exhalation than during inhalation.

This study was a good test of the capabilities of the APD measuring device. Testing confirmed that the APD can detect resistance changes, and that measurements are easy to obtain. Results in this study are generally more consistent than other studies using other techniques [1-11]. The fact that the APD directly measures respiratory resistance, and is not an indirect measurement may be one reason for this consistency. Then, again, our subject population exhibited some homogeneity in age, social class, and racial makeup.

## Conclusions

Nasal strips reduce nasal resistance by about 0.5 cm H_2_O/Lps. Thus, nasal strips do have a measurable effect on nasal resistance. The effect of NDS appears to be equal during exhalation and during inhalation.

The APD can be used to measure nasal resistance, and can detect resistance levels.

The APD can consistently measure oral resistance with either a mask or a mouthpiece.

## Authors' Contributions

LW conducted the testing as an undergraduate student. ATJ provided the APD and mentored LW. All authors have read and approved this manuscript.
